# Study and Validation of Eavesdropping Scenarios over a Visible Light Communication Channel

**DOI:** 10.3390/s17112687

**Published:** 2017-11-21

**Authors:** Ignacio Marin-Garcia, Victor Guerra, Rafael Perez-Jimenez

**Affiliations:** 1 Facultad de Ingenieria en Electricidad y Computacion, Escuela Superior Politecnica del Litoral (ESPOL), P.O. Box 09-01-5863 Guayaquil, Ecuador; 2 Instituto para el Desarrollo de las Telecomunicaciones (IDeTIC), Universidad de las Palmas de Gran Canaria, 35001 Las Palmas de Gran Canaria, Spain; vguerra@idetic.eu (V.G.); rperez@idetic.eu (R.P.-J.)

**Keywords:** visible light communication, network security, data sniffing, eavesdropping, information assurance, communication

## Abstract

The security and privacy provided by Visible Light Communication (VLC) technologies is an area that has been slightly addressed due to the misconception that, since light does not go through solid objects like walls, VLC-based communications cannot be eavesdropped on by outside observers. As an upcoming technology, VLC is expected to be used in multiple environments were, due to radio frequency RF overuse or limitations, RF solutions cannot or should not be employed. In this work, we study the eavesdropping characteristics of a VLC-based communication. To evaluate these concerns, a two-step process was followed. First, several simulations of a standardly used scenario were run. Later on, experimental tests were performed. Following those tests, the results of the simulations and the experimental tests were analyzed. The results of these simulations and tests seemed to indicate that VLC channels can be eavesdropped on without considerable difficulties. Furthermore, the results showed that sniffing attacks could be performed from areas outside the expected coverage of the VLC infrastructure. Finally, the use of the simulation such as the one implemented in this work to recognize places from which sniffing is possible helps determine the risk for eavesdropping that our VLC-based network has.

## 1. Introduction

Visible light communication (VLC) is one of the newest technologies that has been developed for short and middle range data communication. The implementation of VLC is based on the use of the communication band between 380 nm and 780 nm, which is unlicensed. This technology potentially increases the available bandwidth for wireless communication systems, which currently is quite congested at Radio Frequency (RF) bands due to its massive use [1].

The use of VLC as a commercial solution is predicted to have a considerable market penetration in the near future due to the advantages the technology brings. VLC provides a flexible communication channel that, according to the standard [2], allows data transfer rates ranging from 11.67 kb/s to 96 Mb/s. This flexibility is based on the use of three operating modes (PHY I, II and III) of which two (PHY-I and PHY-II) are mandatory to implement. This standard also defines two types of modulation: On–Off Keying (OOK) and Variable Pulse-Position Modulation (VPPM) for the mandated operating modes. VLC uses a different kind of modulation, Color-Shift Keying (CSK), to achieve higher transfer rates for the third operating mode (PHY-III).

Among the multiple benefits that VLC is supposed to bring into the market is the inherited security of the technology. This assumed security is based on the premise that (indoor) light data streams cannot be captured from outside users. Moreover, if a secure channel is required, the Advanced Encryption Standard (AES) with a modified Counter with cipher block chaining message authentication code (CCM*) optional Cryptosystem (AES-CCM*) is specified in the standard. However, no real proof of security has been provided, and no sniffing requirements have been determined.

Reasonable concerns about security have been stated, and some general research into VLC security has been conducted. For example, Mostafa and Lutz in their paper [3] studied the use of null-steering and artificial noise strategies to achieve positive secrecy rates against eavesdropping attacks. In another paper [4], the same authors considered using friendly jamming to secure data transmissions through VLC. Additionally, in [5], Chow, et al. proposed using several LEDs, called intrusion-LEDs, to transmit an interference signal to create a secure area inside which VLC communication was eavesdropping-proof. In [6], Grzegorz Blinowski studied the risk of snooping, jamming and modifying VLC based communications. In the paper [7], Classem, et al. considered the theoretical eavesdropping possibility of VLC based communications through keyholes and door gaps. In [8], a lab test of VLC sniffing using readily available components was performed with positive results. Finally, in a different paper [9], Prasad, et al. compared Ultra-Wide-Band (UWB) and VLC for Data-Intensive and Security-Sensitive Applications. All the presented works begin to consider the feasibility of VLC for secure data communications and try to determine some security and secrecy boundaries for VLC transmissions.

This concern is based on the expected large amounts of data that, in the near future, will be transmitted through VLC networks [10,11]. In addition to this, the information transmitted through VLC, like the one used in geolocation techniques [12,13,14,15,16], could be exploited for criminal activities, since the transmitted data could be of great interest for potential attackers. For the stated reasons, further understanding of the security limitations of VLC and its exploitation should be studied and understood for user protection.

To be able to sniff two critical parameters must be accomplished. The power received by the eavesdropper must be enough to correctly “Read” the signal, and the bandwidth of the signal must be big enough so the signal can be discriminated.

In this work, we proposed the study of eavesdropping on a VLC link. To accurately assess the possibilities of such an attack, simulations and practical experiments were performed. In the simulations, the evaluated channel model was used to determine the amount of received power as well as the signal quality. The test was run using an emitter and receiver over 25 m apart and a telescope to increase the gain of the receiver.

This paper is organized as follows: Section 2 describes the model used for the research. Section 3 presents the results from the simulation (Section 5.1) and from the experiments (Section 3.2). Section 4 puts into context the results of the previous section and examines the implication those results have for VLC-based systems security. Section 5 describes the materials and methods used in both the simulation and experiments. Finally, some conclusions reached in this work are presented.

## 2. Working Model

Indoor VLC links comprise Line-Of-Sight (LOS) and Non-Line-Of-Sight (NLOS) components. LOS components are modeled as the amount of energy radiated by the emitter that directly impacts the receiver. Mathematically, it is calculated as the solid-angle integral of the emitter’s radiation pattern. However, for small photoreceiver area-range relations, the solid angle differential can be approximated by $d\Omega \approx A_{pd}/d^{2}$, yielding as shown in Equation (1):

(1) $$P_{rx}\approx P_{tx}\frac{m+1}{2\pi }\cos^{m}\left(\theta \right)G_{lens}\frac{A_{eff}}{d^{2}},$$

where $P_{tx}$ is the overall optical emitted power, *m* is the directivity coefficient of the emitter (assumed a Lambertian one), θ is the elevation angle, G(ψ) is the lens gain which depends on the relative arrival angle ψ, $A_{eff}$ is the effective area of the receiver, and *d* is the link’s range.

When considering indoor-to-outdoor (or vice versa) links, light travels through windows to reach the receiver. Depending on the type of window, the number of optical interfaces that are crossed vary. However, the simplest scenario implies a single-crystal window and, hence, two interfaces. When traversing an optical interface, light refracts following Snell’s law and an attenuation governed by Fresnel’s equation must be considered. This additional attenuation term can be included in Equation (1), yielding the following expression, Equation (2), in which $L_{w}$ corresponds to the aforementioned two-interfaces Fresnel loss term and $n_{w}$ is the crystal’s refractive index:

(2) $$P_{rx}\approx P_{tx}\frac{m+1}{2\pi }\cos^{m}\left(\theta \right)L_{w}\left(\theta ,n_{w}\right)G_{lens}\frac{A_{eff}}{d^{2}}.$$

**Figure 1:**
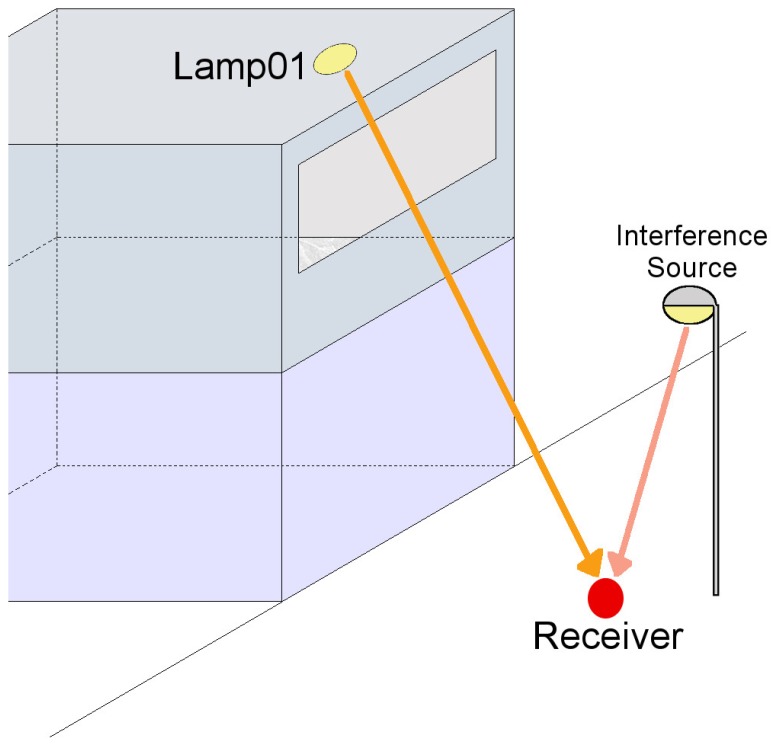
Eavesdropping scenario. The emitter is located on a second floor room and the eavesdropper is located outside. The existence of an interference source, a street lamp, is shown in the figure.

If bandwidth estimation was needed, the impulse response could be approximated by the LOS component plus the first-bounce NLOS component. This final element depends on the receiver’s Field of View (FOV), the scenario’s geometry and the walls’ reflection patterns. For instance, in a scenario like the one shown in Figure 1, the eavesdropper’s bandwidth performance would be much higher than the indoor-located incumbents’ since the FOV limits the incoming contributions to a very narrow region centered on the emitters.

**Figure 2:**
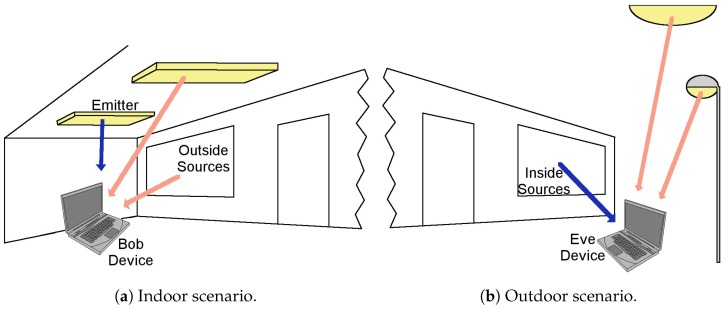
Indoor and outdoor examples of noise sources. In (**a**), two interference sources are present: non Visible Light Communication (VLC) light fixtures and the light coming through a window; in (**b**), two interference sources are present: the non-VLC street light and the sun.

Generally, this situation will be kept in most scenarios, and it could be stated that an eavesdropper in an LOS case will be able to receive a signal with lesser inter-symbol interference (ISI). Nevertheless, there would be considerable received-power constraints and the necessity of high-gain optics, as well as the possibility of suffering sun-based interferences (Figure 2).

In this type of situation, the Signal-to-Noise Ratio (SNR) could be approximated by using Equation (3):

(3) $$SNR\approx \frac{{\left(P_{rx}R\left(\lambda \right)\right)}^{2}}{2q\left[i_{d}+\left(P_{rx}+P_{sun}\varpi \right)R\left(\lambda \right)\right]B+4k_{B}TB\frac{F_{n}}{R_{L}}},$$

where R(λ) is the receiver’s responsivity, *q* is the electron’s charge, $i_{d}$ is the photodiode’s darkness current, $P_{sun}$ is the average sun’s irradiance, ϖ is the scenario’s albedo, $k_{B}$ is Boltzmann’s constant, *T* is the receiver’s temperature, *B* is the noise bandwidth, and $F_{n}$ and $R_{L}$ are the amplifier’s noise figure and gain, respectively.

Note that the sun generates both shot noise and offset level. This offset could be high enough to saturate the photoreceiver. Therefore, the receiver’s electrical topology must ensure a proper dynamic range.

## 3. Results

To validate the premise that VLC-based communication can be eavesdropped on from outside the premises as long as there is an “opening”, such as a window, two tests were conducted, a simulated one (Section 3.1) and an experimental one (Section 3.2).

### 3.1. Simulation Results

**Figure 3:**
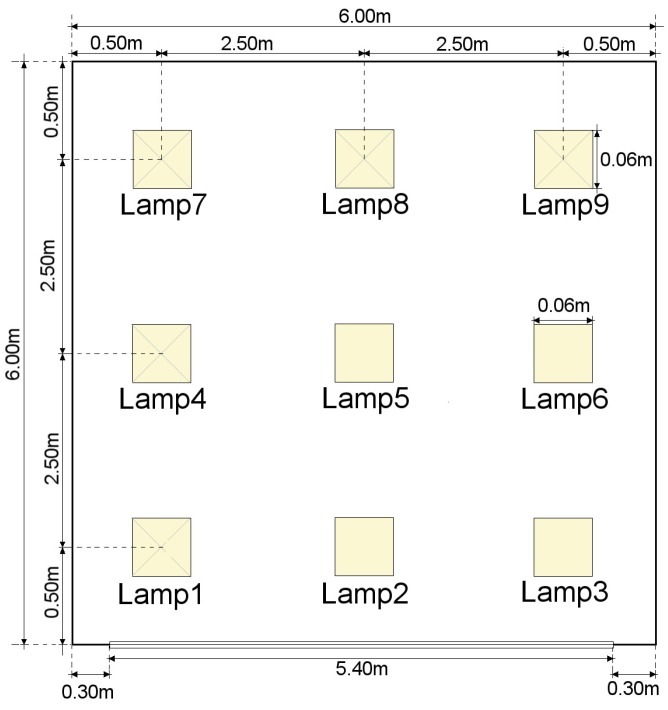
Room Configuration. In the scenario, the emitters are located in a room 6 m wide by 6 m deep by 3 m high. In the scenario’s room, there are nine luminaries with a power output of 12 W per luminary. Each luminary is composed of 100 individual LEDs and each luminary is a square one.

For the environment simulation. a basic scenario proposed in the literature [17,18,19] was used. Distribution of luminaries and room configuration is shown in Figure 3. Further details can be found on Section 5.1.

**Figure 4:**
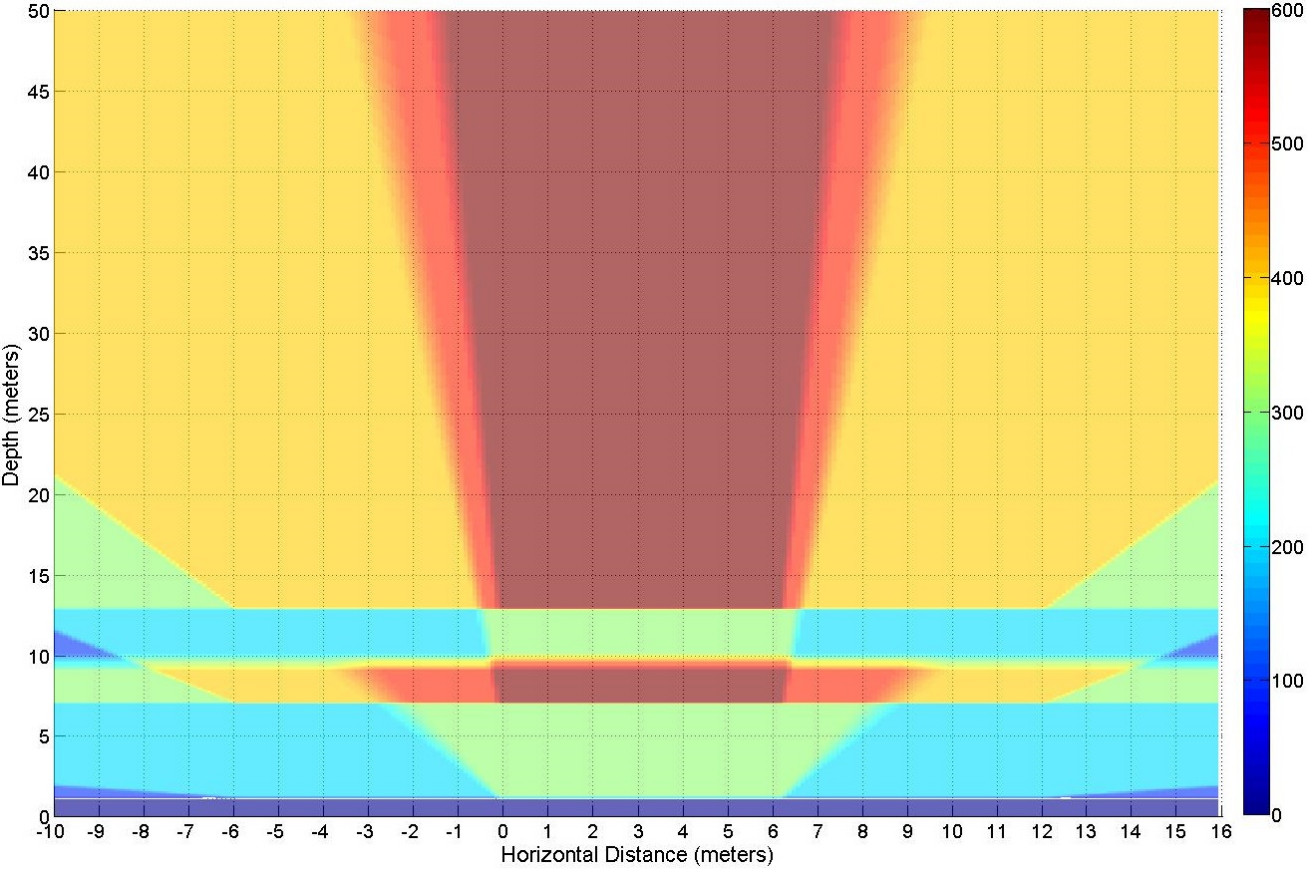
Number of emitters (LEDs) viewed from the target area. The observation area is 50.00 by 26.00 m and is located at the height of 0.00 m. The window being observed is at 4.20 m of height and located between the 0.00 to the 6.00 m in the horizontal axis.

The definition of potential attack zones was based on pure geometrical considerations and assuming an LOS approach. A slice-based graph will be presented, showing how the number of available lamps varies depending on the position of the eavesdropper. The result of this first step, number of LEDs seen by the receiver using an LOS approach, is shown in Figure 4. It should be noted that, in the first 20 cm closest to the “window”, the number of LEDs viewed is zero.

The results of the simulation: number of LEDs seen with a $180^{\circ }$ FOV and $20^{\circ }$ FOV, power received ($P_{i}$), and bandwidth (BW)), of the second step are listed on Table 1. The values corresponding to each of the columns are:

- Position follows the 〈width,depth,height〉 format and is expressed in meters.
- There are two “Viewed LEDs” columns:
  - The first column represents the number of LEDs that can be viewed from that position having an FOV of $180^{\circ }$. This variable represents the number of individual LEDs that a single attacker may observe from that position by eye.
  - The second column represents the number of LEDs that can be viewed from that position having an FOV of $20^{\circ }$. This variable represents the number of LEDs that an attacker observes using some help that restricts the FOV, down to $20^{\circ }$, but may increase the receiver gain such as the employment of the telescope used for the experiments (Section 3.2).
- The power value, in the fourth column, is in mW and represents the receiver power in that position with an FOV of $20^{\circ }$ and no extra gain.
- The bandwidth in MHz and, as in the previous value, it represents the bandwidth obtained with an FOV of $20^{\circ }$.

**Figure 5:**
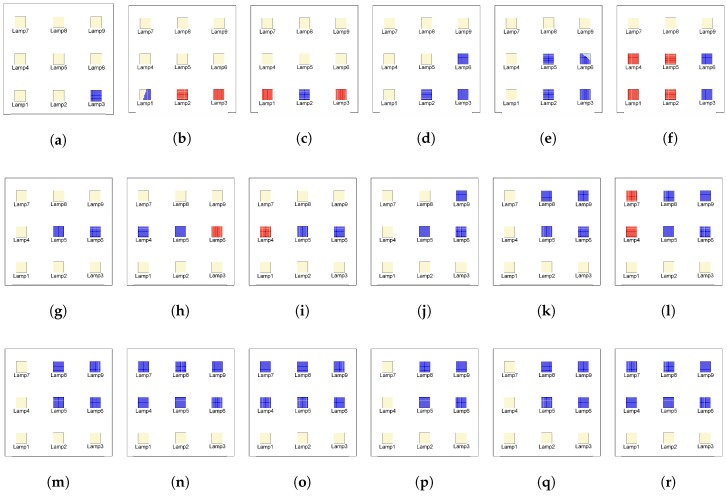
LEDs viewed from the receiver’s position: (**a**) S1; (**b**) S2; (**c**) S3; (**d**) S4; (**e**) S5; (**f**) S6; (**g**) S7; (**h**) S8; (**i**) S9; (**j**) S10; (**k**) S11; (**l**) S12; (**m**) S13; (**n**) S14; (**o**) S15; (**p**) S16; (**q**) S17; and (**r**) S18 as listed in Table 1. Blue points represent the LEDs viewed by the receiver with an FOV of $20^{\circ }$. Red points represent the LEDs viewed by the receiver, in addition to the blue ones, when the receiver has an FOV of $180^{\circ }$.

Additionally, to clarify which LEDs were being seen by the receiver at each of the points selected, and shown in Table 1, those LEDs were plotted as seen in Figure 5. The blue points in Figure 5 represent the LEDs viewed by the receiver using an LOS approach and an FOV of $20^{\circ }$. In addition to those blue points, if the receiver had an FOV of $180^{\circ }$, the LEDs represented by the red points would also be seen by the receiver.

### 3.2. Experimental Results

For the experimental part of the work, a similar simulation was performed using an emitter located on the second floor of the IDeTIC building at the “Universidad de Las Palmas de Gran Canaria”. Of the possible points from which the eavesdropping was tried, four were chosen. The distances, direct and vertical of those positions, are listed in Table 2.

**Figure 6:**
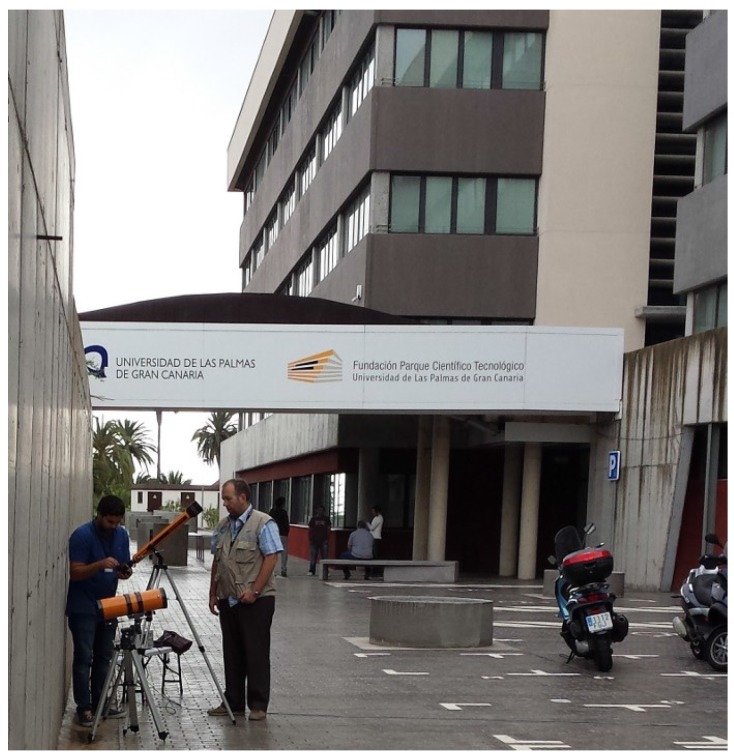
Assembly used to eavesdrop on the VLC channel from position T1.

**Figure 7:**
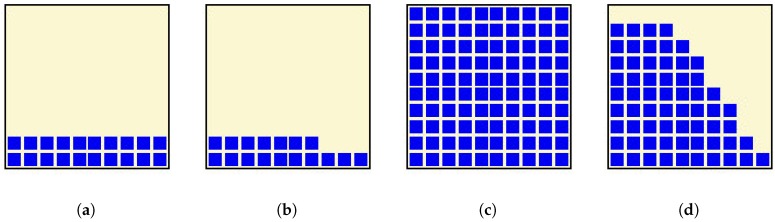
Representations of the expected LEDs viewed from the different locations as simulated: (**a**) at T1; (**b**) at T2; (**c**) at T3; and (**d**) at T4 as defined in Table 2.

**Figure 8:**
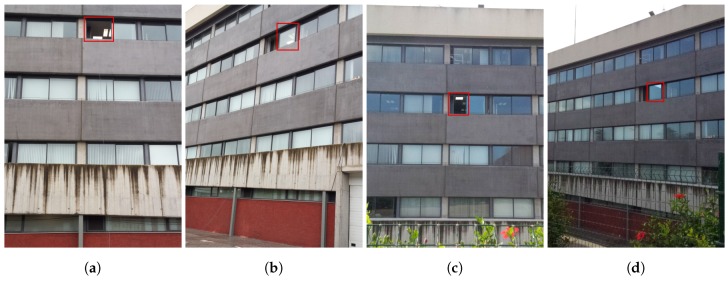
Pictures of the target emitter from the diferent locations where the sniffer was positioned: (**a**) T1; (**b**) T2; (**c**) T3; and (**d**) T4 as defined in Table 2. In (**b**,**d**), it can be observed that the double panel window is between the emitter and the receiver. In (**a**,**c**), the receiver has a clear and direct view without obstacles of the emitter; and the window in which the emitter can be observed is highlighted in red.

The simulated power received in the positions shown on Table 2, the expected number of LEDs viewed from those positions as well as the tested power received in those areas are shown in Table 3. Power values are in mW, and viewed LEDs are a percentage of the total expected emitting LEDs on the lamp. In our case, a receiver as shown in Figure 6 was used, and a ten by ten matrix of LEDs was assumed. Furthermore, in Figure 7, it can be seen which part of the lamp could be seen from the different locations specified in Table 2 according to the computer simulation. The corresponding pictures are shown in Figure 8.

**Figure 9:**
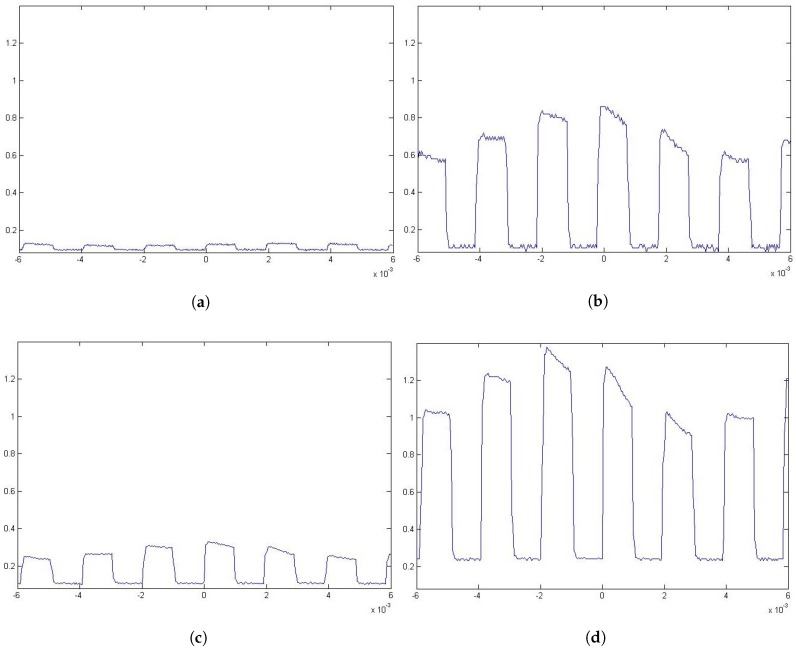
Captures of the signal from diferent locations: (**a**) at T1; (**b**) at T2; (**c**) at T3; and (**d**) at T4 as defined in Table 2. The emitted signal used an On-Off keying with non return to zero (OOK-NRZ) modulation scheme at a 512 bps data rate.

**Figure 10:**
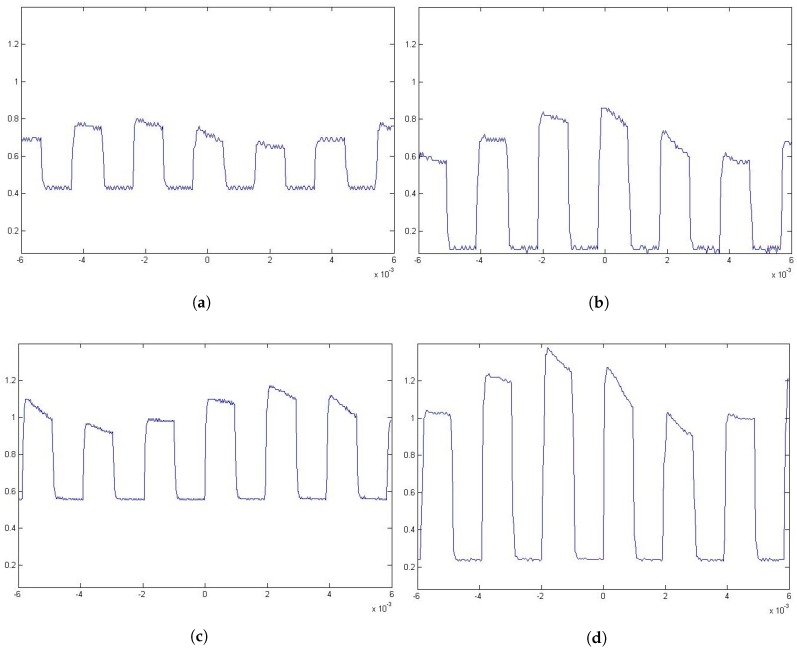
Subfigures (**a**,**b**) are measures taken from location T2 being (**a**) a case in which the windows are present between the emitter and the receiver and (**b**) a case in which the windows are not present between the emitter and the receiver. Subfigures (**c**,**d**) are measurements taken from location T4 being (**c**) a case in which the windows are present between the emitter and the receiver and (**d**) a case in which the windows are not present between the emitter and the receiver. In all cases, the positions are as defined in Table 2. Vertical axes denote power in nW and the horizontal axes denote time in s.

During the test, the channel bandwidth could not be measured due to the hardware limitations. Nevertheless, in all cases, as it can be observed in Figure 9, the signal could be captured relatively clearly in all cases. Even when windows are between the emitter and the receiver, as in the cases shown in Figure 8b,d, the signal can be recognized, as is shown in Figure 10.

In Table 4, the results of the sniffing/eavesdropping experiment can be observed. Values for the background interference level (**BIL**); signal mean power (**SMP**); electrical SNR and channel capacity are provided. As in all of this paper, the positions are identified by T1, T2, T3, and T4 names corresponding to the positions from which the pictures of Figure 8 were taken and whose distances are shown in Table 2. In the cases of positions T2 and T4, two scenarios were considered: one in which the windows panes were between the emitter and the receiver (with windows) and one in which they were not as in positions T1 and T3.

## 4. Discussion

Generally speaking, up until recently, VLC-based solutions were considered a secure way to communicate information since “light could not go through walls”. However, when we consider that most habitats have some window, due to design or regulatory requirements, we have to start thinking that, at least, some information leakage will occur even when some interference sources are emitting to obscure the signal, as has been proposed in [4,5]. These interference sources could be circumvented by directing the focus of the eavesdropper to the real emitter.

A first approach to determine the risk of information leakage in VLC channels can be made through a simulation of the environment. In our case, the simulation (Section 3.1) shows that, even if the more significant amount of leakage is located directly in front of the window, there are significant leakages on the sides, and the higher power density happens to be not in the closest locations but the middle range ones as shown in Figure 4 and Table 1.

However, when we observe Table 1, we note that when we are close to the emitters, such in positions S1 and S3, we get a higher bandwidth (3.76 and 8.16 GHz, respectively). Opposite to the previous situation, we have position S2, located between S1 and S3, where the bandwidth available (24.66 MHz) is smaller than the other two (3.76 and 8.16 GHz). At the same time, the power density received at S2 is the third largest (30.44 mW) after the one at S3 (42.95 mW) and the one at S5 (41.42 mW).

It can also point out that, even if in the first position, S1, we get a higher bandwidth than in almost any other of 16 tested locations, and the power density received is smaller than 12 of those who happen to be farther from the sources. The reason for this is the interference that the multiple sources, which emit the same signal synchronously, create. Therefore, the bandwidth limitation should be considered when eavesdropping since the available bandwidth may make it impossible if it is smaller than the bandwidth used in such communication.

The use of multiple spatially separated sources has been proposed to improve the communication link data rate of VLC [20,21] forming visual Multiple-Input Multiple-Output (MIMO)systems. This approach aims to give protection to the data since these coexisting signals can be considered as interference. This method is similar to procedures successfully applied for RF systems, such as in [22]. However, in the case of VLC eavesdropping techniques, the listener could still attack a MIMO-based link by using image-forming optics and an adequate receiver. Due to the theoretical feasibility of this kind of attack in MIMO systems, the scenario’s geometry could lead to an impracticable situation due to the closeness of the light sources. In this work, only synchronized multi-LED emitters have been considered.

An important aspect when considering the position is the aperture of the receiver. This aperture will influence the outcome significantly the eavesdropping as well as the selection of the location from where the eavesdropping will be conducted. As can be observed in Figure 5, the site of the receiver changes the list of viewed emitters and therefore the outcome of the attack.

All of these effects should be considered when considering eavesdropping for either defensive purposes or offensive (i.e., penetration tests) ones. For this reason, simulating the environment is an adequate tool for securing the environment to secure. To validate this latest premise, we executed a simulation and several experiments in a known location. As expected, the simulation generated areas of interest from which eavesdropping occurred had a higher possibility of success. The chosen locations are listed in Table 2.

Of the four chosen locations, from T1 to T4, it was expended, as shown in Table 3, that the T3 position, having a more direct line of sight to the emitter as the second shortest distance (19.00 m), had the best signal. This preconception happened to be true, and the power density that reached our receiver was twice as high as any of the other positions selected.

The location with the expected better received power density as T1 as it was the closest one to the emitters. When tested, this proved wrong, T4 being the place with the second better-received power density. Since, in the simulation, there was a difference smaller than 4.00%, and the percentage of the emitter viewed by the receiver was higher at the T4 position (64% vs. 20%), measurement errors may justify the discrepancy between expected and tested values.

The power received by the locations located “diagonally” to the emitter, T2 and T4, had a small difference in the simulations—less than 7%. However, this difference increases up to 25% in the tests, as can be seen in Table 3. At the same time, as is shown in Table 4, the background interference level obtained during the test increases, as can be expected, with distance making the signal harder to interpret.

When we compare the results of the simulated scenario and the test scenario, it can be observed that, in the case of the experimental one, the power that reached the receivers in the different positions was an order of magnitude than the expected received value (Table 3). The reason for this incoherence may be the factors that were evaluated. These factors didn’t include, for example, the lamp diffuser or the optoelectrical conversion on the emitter side. These unaccounted losses can explain the difference in magnitudes from the expected received power and the actual received power. Moreover, aiming at a small target and then using a photodiode to capture the signal proved challenging and aiming errors were common. Nevertheless, higher data rate eavesdropping requires higher received power and better SNR as shown in Table 4. To obtain those demanded SNR values, the attacker, as it was in our case, can play with the focus and gain of the sniffing device and, in general, the attacker can obtain a better SNR than a regular user so the result will benefit the eavesdropper most of the time from the calculated channel capacity, as observed in Table 4, and the fact that an outdoor located eavesdropper, with a powerful enough optical device, could reduce the incoming energy contributions from interference sources obtaining a purer LOS with the base station. This will conclude that eavesdroppers would generally have better channel capacity than system legit users.

The results seem to validate that it is possible to eavesdrop on a VLC-based channel from outside the premises, as it was previously accepted. Even if the values obtained by the experimental tests (Table 1) are not the same as the ones obtained from the simulations (Table 1) by an order of magnitude, the general distribution of the results is similar in both cases. The differences in values may be attributed to unmeasured effects such as the optoelectrical conversion in the emitter, the use of diffusers on the lamp or multiple aiming errors that decreased the received power. These results, in turn, ratify the usefulness of using simulations for select areas, or positions, of interest from which the attack is performed. These elected positions increase the chance of capturing a clear signal and may be located outside the expected attacker’s area. In the case of our experimentation, one such location was diagonally from the window (T4 in Table 2), which is mostly outside the view from the inside, but provides a clear and powerful enough signal (Table 4 and Figure 9d) with or without window panels in between (Figure 10c,d).

## 5. Materials and Methods

### 5.1. Simulation Materials and Methods

**Figure 11:**
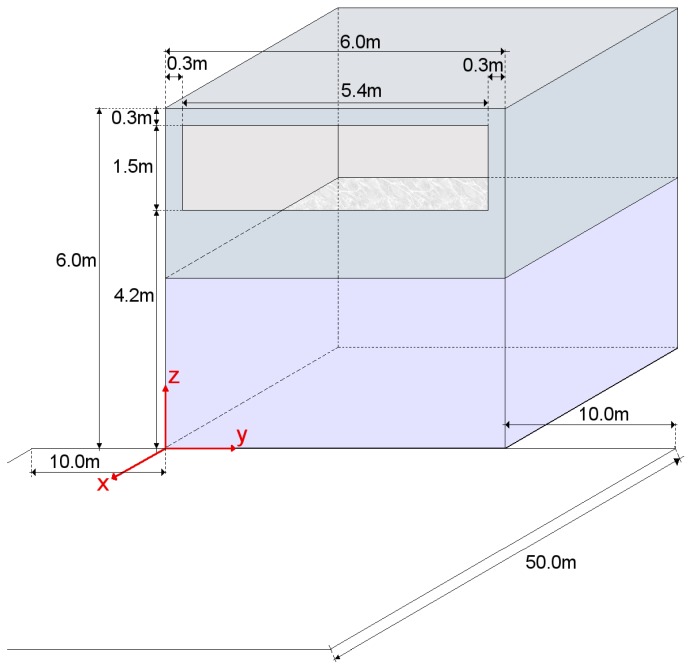
Simulation configuration. In the scenario, the emitters are located in a room 6 m wide by 6 m deep by 3 m height. In the scenario’s room, there are nine luminaries with a power output of 12 W per luminary. Each luminary is composed of 100 individual LEDs and each luminary is a square one.

A standard scenario [17,18,19] was modified to consider an outdoor eavesdropping. The room was changed by the addition of a window 5.40 m wide by 1.50 m hide located 1.20 m from the floor and 0.30 m from the side walls and ceiling. The room was located on a second floor as shown in Figure 11 so the window was at the height of 4.20 m from the street. The expected location for the eavesdropper considered the street and parking area located on the front of the building where the emitters were located. All this area was defined as the target area from which an attack was possible, and it was delimited in an area of 26.00 m wide by 50.00 m long.

For the simulations, a multiple step approach was considered. The first step was determining in which points on the target area more emitters were visible. As stated, the receiver target area was a 26.00 by 50.00 m one. This approach allowed for the receivers to be located up to 10.00 m from the side of the room (or 10.30 m from the window’s edge).

The second phase of our simulation approach was choosing several locations, based on the presence or absence of LOS to multiple emitters and calculating the power received and the bandwidth available at those points. The selected points for this second phase tried to take into account the variances shown in phase one that included differential received power and number of emitters so the effect in the bandwidth could be understood. The bandwidth available at a point was calculated by applying Equations (4)–(7):

(4) $$H\left(0\right)=\int h(t)dt\equiv \sum_{i=1}^{N}P_{i},$$

(5) $$\begin{array}{cc} \bar{T}=\int_{t}t\frac{h(t)}{H(0)}dt & \equiv \sum_{i=1}^{N}t_{i}\frac{P_{i}}{H(0)}, \end{array}$$

(6) $$\begin{array}{cc} \tau_{rms} & =\sqrt{\int {\left(t-\bar{T}\right)}^{2}\frac{h(t)}{H(0)}dt}\equiv \sqrt{\sum_{i=1}^{N}{[\left(t_{i}-\bar{T}\right)}^{2}\frac{P_{i}}{H(0)}}]\\ & =\sqrt{\sum_{i=1}^{N}\left[{\left(t_{i}-\sum_{i=1}^{N}t_{i}\frac{P_{i}}{\sum_{j=1}^{N}P_{j}}\right)}^{2}\left(\frac{P_{i}}{\sum_{i=1}^{N}P_{i}}\right)\right]}, \end{array}$$

(7) $$BW\approx \frac{1}{5\tau_{rms}}.$$

In Equations (4)–(7), *N* is the number of lamps with LOS from the receiver point of view, $P_{i}$ is the power received from that lamp and *t* is the time of arrival of the same signal.

As stated, each of the lamps was an array of 100 LEDs emitting a total of 12 watts, so each LED emitted 0.12 watts of power. For the computer simulations, the receiver had a 20${}^{\circ }$ FOV, and no optical or electrical gain was applied.

To choose the points from which the eavesdropping was tested, several constraints were considered:

- The lower the FOV of the lenses, the higher the receiver power. Therefore, reducing the FOV through optical means, using a telescope in our case, increased the receiver power.
- The higher the FOV, the higher the background noise. Therefore, decreasing the FOV would also decrease the background noise and improve the acquired signal.
- The higher the FOV, the higher the probability of receiving light from different sources. In our case, since the network access points emit simultaneously the same information, the receiver will be affected by ISI. On the other hand, if the access points are part of a cellular network, an SINR could be defined on the received signal using Equation (8):
  (8) $$SINR=\frac{{\left(P_{tx}H\left(0\right)R\left(\lambda \right)\right)}^{2}}{\sigma_{th}^{2}+\sigma_{sh}^{2}+\sum_{i\in I}{\left(P_{i}H_{i}\left(0\right)R\left(\lambda \right)\right)}^{2}}.$$
- The more directive the emitters, the lower the received power due to the radiation pattern’s thin shape. For this work, general Lambertian emitting patterns were used.
- The longer the distance, the higher the turbulence effect. Moreover, due to the signal propagation characteristics, the receiver power decreases with the distance. Even with optical gains, the turbulence affects the communication channel, so, for example, time of the day will affect the communication. (i.e., higher turbulence during the daytime due to thermal currents.).
  It should be noted that, contrary to the RF case where the power of the signal decreases with the square of the distance, as shown in Equation (9), in the VLC case, the power falls faster, as shown in Equation (10), due to the optoelectric conversion:
  (9) $$\begin{array}{cc} P_{elec}^{RF} & \propto P_{Tx}G_{Tx}G_{Rx}{\left(\frac{\lambda }{4\pi d}\right)}^{2}\\ P_{elec}^{RF} & \propto d^{-2}, \end{array}$$
  (10) $$\begin{array}{cc} P_{elec}^{VLC} & \propto {\left[P_{Tx}S\left(\theta \right)G_{lens}\frac{A_{eff}}{d^{2}}R\left(\lambda \right)\right]}^{2},\\ P_{elec}^{VLC} & \propto d^{-4}. \end{array}$$
- The channel capacity was large enough for the eavesdropping. The channel capacity was calculated using Equation (11):
  (11) $$C=BW\;log_{2}\left(1+\frac{S}{N}\right).$$

All the concerns as mentioned earlier should be taken into account at the moment of choosing the points from which the sniffing attack will be held.

### 5.2. Experimental Materials and Methods

To validate the different scenarios and simulations, we did several real-world tests by creating a test bed using a commonly used illumination device. The characteristics of the emitter device are shown in Table 5.

**Figure 12:**
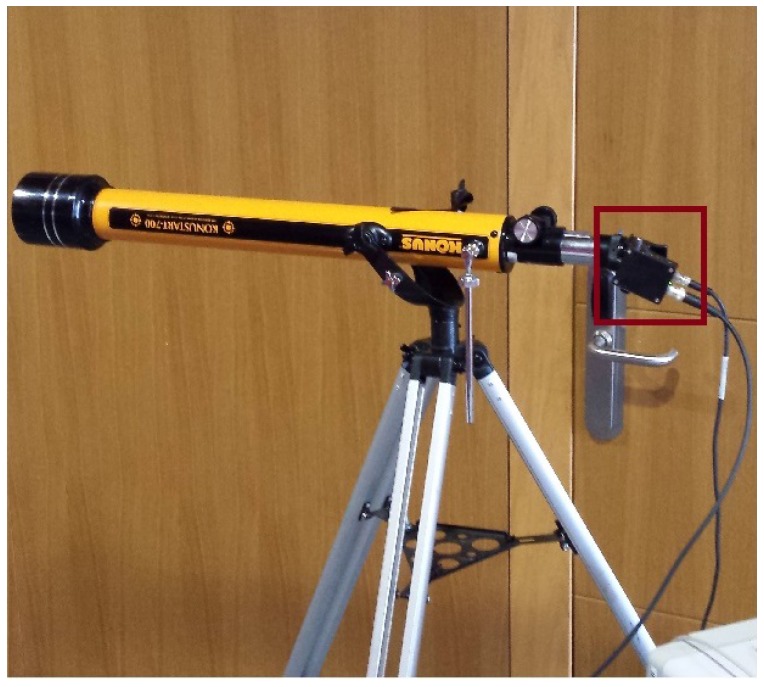
Receiver Assembly. The photodiode used for the data capture is highlighted in red.

The characteristics of the receiver device can be seen in Table 6. An image of the assembly is shown in Figure 12.The photodiode used for the capture is highlighted in red.

On the receiver side, a telescope was used to increase the gain of the receiver. The gain from the telescope ($G_{lens}$) was calculated using Equation (12). The telescope had a lens with a diameter (ϕ) of 60 mm${}^{2}$. The area in which the light was projected had a diameter of 16 mm${}^{2}$. This configuration resulted in an approximated gain ($G_{lens}$) of 14.0625:

(12) $$G_{lens}\approx \frac{A_{Lens}}{A_{projected}}\approx {\left(\frac{\phi_{lens}}{\phi_{projected}}\right)}^{2}.$$

The total receiver’s gain included the gain from the telescope ($G_{lens}$), the responsivity (*R*) of $0.3\frac{A}{W}$, and an electronic gain ($G_{elec}$) of 70 dB. When Equation (13) was applied, we got an approximated total gain (*G*) of $13.34\frac{V}{mW}$:

(13) $$G=G_{lens}\cdot R\cdot G_{elec}.$$

Four target positions at different heights and distances were selected for the test. The distances and height for each one of the locations are shown in Table 2. As can be observed, the positions were selected to emulate possible scenarios such as eavesdropping from a parking space or from outside the premises. The view of the emitter from the different locations as well as the general assembly used are shown in Figure 8. The distances were used to calculate the theoretical received power at each position and to compare those values with the ones obtained in the experiments as shown in Table 3.

## 6. Conclusions

In this work, we proposed the study of eavesdropping on a VLC link. To assess such an attack, simulations were performed. To validate the results from the simulations, practical experiments were performed.

The study proved the validity of using geometrical considerations to define potential attacks zones. This study served as the base to choose, from those potential attacks zones, the ones that seemed more adequate. Additionally, the simulations showed that even if the more significant amount of leakage was located directly in front of the window, there are significant leakages on the sides, and the higher power density happened to be not in the closest locations but in the middle range ones. The interference between sources decreased the bandwidth available for eavesdroppers. However, this issue affected both legit and non legit users so it can be minimized when a VLC system is implemented.

An important aspect was the aperture of the receiver. This aperture will influence the outcome significantly, as well as the selection of the location from where the eavesdropping will be conducted. However attackers can modify their receiver’s aperture more freely than legit users.

Although the simulations provide a good starting point, validating those results with experimental testing proved to be a necessity. Due to measurement and aiming errors, the resulting values vary significantly. However, the simulations and the experimental test proved that eavesdropping a VLC link is possible and that the attacker is less limited than previously expected.

In turn, the simulations are useful for selecting areas, or positions, of interest from which the attack may be carried out. These positions increase the chance of capturing a clear signal and may be located outside the expected attacker’s area.

The results seem to validate that it is possible to eavesdrop on a VLC-based channel from outside the premises, as it was previously accepted. Furthermore, this is exploited since the received power can be incremented while the noise can be decreased through optical means.

## Figures and Tables

**Table 1:** Simulation results.

Position	Coordinates (*x*, *y*, *z*)	Viewed LEDs		Power (mW/dBm)	Bandwidth (MHz)
		FOV $180^{\circ }$	FOV $20^{\circ }$		
S1	〈1.50,−9.00,0.00〉	100	100	5.14/7.11	3760.00
S2	〈1.50,−0.30,0.00〉	254	54	30.44/14.83	24.66
S3	〈1.50,5.00,0.00〉	300	100	42.97/16.33	8160.00
S4	〈8.00,−9.00,0.00〉	300	300	15.22/11.82	44.13
S5	〈8.00,−4.00,0.00〉	400	400	41.42/16.17	52.89
S6	〈8.00,5.00,0.00〉	600	200	28.13/14.49	30.65
S7	〈12.00,−9.00,0.00〉	200	200	4.96/6.95	22.17
S8	〈12.00,−0.30,0.00〉	300	200	9.61/9.83	69.64
S9	〈12.00,5.00,0.00〉	300	200	8.62/9.36	73.78
S10	〈15.00,−9.00,0.00〉	300	300	5.35/7.28	14.50
S11	〈15.00,−4.00,0.00〉	400	400	9.80/9.91	50.20
S12	〈15.00,5.00,0.00〉	600	400	10.92/10.38	51.13
S13	〈25.00,−9.00,0.00〉	400	400	2.70/4.31	48.91
S14	〈25.00,−0.30,0.00〉	600	600	5.15/7.12	48.83
S15	〈25.00,5.00,0.00〉	600	600	4.97/6.96	49.19
S16	〈45.00,−9.00,0.00〉	400	400	0.65/−1.87	6.42
S17	〈45.00,−4.00,0.00〉	400	400	0.69/−1.61	6.70
S18	〈45.00,5.00,0.00〉	600	600	1.05/0.21	48.41

FOV: Field of View.

**Table 2:** Sniffing positions.

Position	Coordinates	Distance (m)	Δh(m)	Azimuth
T1	〈13.90,−0.90,−10.65〉	17.53	10.65	$37.50^{\circ }$
T2	〈13.90,11.10,−10.65〉	20.73	10.65	$35.50^{\circ }$
T3	〈19.60,−0.90,−8.25〉	21.28	8.25	$28.00^{\circ }$
T4	〈20.40,15.10,−7.85〉	26.57	7.85	$20.00^{\circ }$

**Table 3:** Sniffing results.

Position	Simulated Power (mW/dBm)	Viewed LEDs	Tested Power (mW/dBm)
T1	0.9876/−0.0542	20%	0.0014/−28.5387
T2	0.8684/−0.6128	17%	0.0031/−25.0864
T3	1.1005/0.4159	100%	0.0087/−20.6048
T4	0.9251/−0.3381	64%	0.0044/−23.5655

**Table 4:** Sniffing results.

Position	BIL (nW/dBm)	SMP (nW/dBm)	Electrical SNR (dB)	Channel Capacityx (Gbps)
T1	4.8550/−53.1381	1.3692/−58.6353	17.1691	14.2771
T2 ${}^{†}$	2.2186/−56.5392	1.4142/−58.4949	20.4710	0.3774
T2	5.9669/−52.2425	3.0506/−55.1561	21.7965	0.4016
T3	5.6765/−52.4592	8.7199/−50.5949	20.5340	2.5841
T4 ${}^{†}$	29.1200/−45.3581	23.6920/−46.2540	19.5748	0.2466
T4	13.5230/−48.6893	44.3630/−43.5298	19.9667	0.2515

Note: The symbol ${}^{†}$ denotes measures in which a double panned window was between the emitter and the receiver.The channel capacity values were obtained using the theoretical channel bandwidth and without taking into account the bandwidth of the emitter and the receiver. If those values were taken into account, the channel capacity would decrease. BIL is the background interference level, SMP is the sygnal mean power, and SNR is the signal to noise ratio.

**Table 5:** Emitter characteristics.

Parameters	Value
Electrical Power	40 W
$R_{a}$	>80
Emission angle	120${}^{\circ }$
Emission Frequency	512 Hz
Modulation Scheme	OOK-NRZ

**Table 6:** Receiver characteristics.

Parameter	Value
Lens ϕ	60 mm
Photodiode ϕ	5 mm
Electrical Gain	70 dB

## Abbreviations

The following abbreviations are used in this manuscript:

bps	bits per second
AES	Advanced encryption Standard
CBC-MAC	Cipher Block Chaining Message Authentication Code
CCM	Counter with CBC-MAC
CSK	Color-Shift Keying
FOV	Field of View
ISI	Inter-Symbol Interference
LED	Light Emitting Diode
LOS	Line-of-Sight
MIMO	Multiple-Input Multiple-Output
NLOS	Non Line-of-Sight
NRZ	Non-Return-to-Zero
RF	Radio Frequency
OOK	On–Off Keying
SNR	Signal-to-Noise Ratio
UWB	Ultra-Wide Band
VLC	Visible Light Communication
VPPM	Variable Pulse-Position Modulation
